# The tragedy of the biodiversity data commons: a data impediment creeping nigher?

**DOI:** 10.1093/database/bay033

**Published:** 2018-04-09

**Authors:** Nora Escribano, David Galicia, Arturo H Ariño

**Affiliations:** Universidad de Navarra, Department of Environmental Biology, Biodiversity and Environmental Quality Data Analysis Group, 31008, Pamplona, Spain

## Abstract

Researchers are embracing the open access movement to facilitate unrestricted availability of scientific results. One sign of this willingness is the steady increase in data freely shared online, which has prompted a corresponding increase in the number of papers using such data. Publishing datasets is a time-consuming process that is often seen as a courtesy, rather than a necessary step in the research process. Making data accessible allows further research, provides basic information for decision-making and contributes to transparency in science. Nevertheless, the ease of access to heaps of data carries a perception of ‘free lunch for all’, and the work of data publishers is largely going unnoticed. Acknowledging such a significant effort involving the creation, management and publication of a dataset remains a flimsy, not well established practice in the scientific community. In a meta-analysis of published literature, we have observed various dataset citation practices, but mostly (92%) consisting of merely citing the data repository rather than the data publisher. Failing to recognize the work of data publishers might lead to a decrease in the number of quality datasets shared online, compromising potential research that is dependent on the availability of such data. We make an urgent appeal to raise awareness about this issue.

## Data sharing: opportunities, limitations and future

Over the last decade, many biodiversity informatics initiatives at global, regional and local scales have emerged with a clear goal: to compile and share data, making science open worldwide (1). Ideally, data freely available on the internet offer a vast range of potential uses that could foster advances in biodiversity research and better support for decision-making (2–4). Current funding and publishing practices increasingly call for publication of datasets, and most researchers are assuming that publishing the data is therefore not only desirable, but mandated. Indeed, a roadmap to eventually institutionalize the publication of datasets has been proposed (5) and many challenges of such a cultural change have already been identified (6). However, making data accessible, understandable and truly reusable remains a challenge (7, 8): data cannot be shared uncritically. Data need to be mobilized according to solid standards and in a structured way (2), and their quality and that of their associated metadata should be as good as possible (9). Both conditions have additional costs that not all the researchers may be willing to afford.

Concerns about data reliability and usability, the how and why of their publication, have repeatedly emerged in the literature (4, 7–9). Huang *et al.* (13) and Tenopir (12) researched the willingness to publish data among researchers. They found that the majority recognized that sharing data was necessary, and most were willing to do that after publication. However, lack of credit and insufficient time to publish data were identified as obstacles preventing such sharing. As a result, it is common for most data not to remain accessible after the publication of analyses based on them (14), often preventing reproduction and independent verification (7).

Such obstacles could become less critical if datasets are used as a citable reference (10). Data sharing could then become an attractive option for data publishers, potentially enhancing the researcher’s career and influence. Researchers are increasingly realizing that the lifecycle of their research should not end once data have been analysed and the corresponding results are released through a scholarly publication. Datasets themselves should also be considered as a research output (12, 6) and therefore should be peer-reviewed and citable (10).

## How to give recognition to data publishers?

Giving proper credit to data providers is a known, increasingly recurring issue in many fields (3, 14–17). With the advent of the Open Access movement, journals focused on fulfilling the need to publish datasets and the associated metadata have flourished (18). In 2011, *Pensoft Publishers* introduced the data paper, a new type of article that was ‘a scholarly publication of a searchable metadata document describing a particular online accessible dataset or a group of datasets’ (19). Other publishers have then released dataset-describing articles, such as Nature’s ‘data descriptor’ or Elsevier’s ‘data article’. On the other hand, persistent online identifiers now greatly facilitate tracking data sources. Many data repositories worldwide now use the Digital Object Identifier (DOI; 20) to identify and credit the datasets they provide.

However, despite all these tools and initiatives potentially facilitating scholarly recognition of the effort put forth by data authors, citing data correctly is still a pending task for most researchers (21). Many researchers may find the work of making data publicly available unappealing if it is a time- and effort-consuming process that may ultimately go unnoticed and unacknowledged, in a context where scholarly proficiency, a critical factor in researchers’ careers, is largely, if incorrectly, proxied in terms of the impact factor of the publishing medium rather than the individual achievement (22, 23). A similar plight has already been observed in the field of Taxonomy, which necessarily underpins biodiversity research. The work of taxonomists has allegedly been undermined by the generally low impact of their research, leading to grim career advancement opportunities and low funding prospects that might have contributed to their shortage (24, 25). While not the sole reason for the current taxonomic impediment (26, 27), we should expect a similar ‘data impediment’ forming when contributing factors are in place.

## From theory to practice: the global biodiversity information facility data

The Global Biodiversity Information Facility (28) is, by far, the largest resource for readily-accessible biodiversity data. Since its launch in 2001, the growing number of biodiversity datasets shared through its portal has facilitated global research addressing increasingly urgent threats to biodiversity (1, 29). An obvious question for a data publisher (and a heads-up for a data user) is whether the research based on data shared through GBIF is properly crediting the data providers and the data used can be traceable. We explored this practice at pilot scale by recording how articles that used data published through GBIF cited the source of the data.

The GBIF Public Library is curated by a group of scientists that have identified and tagged at least 4533 scientific articles that use data retrieved through the GBIF portal, to assess how such data contribute to research. We randomly selected and analysed 100 papers from the set (date of access 11/05/2017, see Supplementary Appendix S1), published in 51 journals from 2007 to 2017. For each paper, we recorded whether the original data providers were cited or acknowledged in the article, or the GBIF portal was listed as the source (sometimes even as merely a mention in the text) without including in the reference list the actual dataset being used.

Only 2% of the sample papers cited the original dataset in the article’s Reference Section, while a further 4% did so in the Supplementary Material (see Figure 1 in Box 1). Another 2% of the papers used one of the recommended citation practices by GBIF, that is the DOI of the download that points to all the datasets included in the recordset. As for the GBIF data portal, only 18% of the papers cited GBIF itself in the reference list. All other papers (74%) merely mentioned GBIF inline within the text (but not in the references). Considering the desirability citation practices displayed in the Box 1, 88% of the papers failed to cite the datasets in such a way so as to eventually allow them to be traced back to their original source.

Box 1. Desirability map of citing practices

A well-established procedure for attributing data sources to their data publishers does not yet exist (1) despite its obvious advantages to promote recognition and, therefore, data publishing. Here, we arrange how datasets are cited in the literature according to what is cited (columns) and where in the paper the citation occurs (rows). This arrangement results in a rank representing how likely they would result in a traceable, accountable cite. Citable items could be the datasets, either directly or through its data paper; the reference to the downloaded file containing the recordset selected, normally represented by a DOI of the download; or the repository or institution from which the recordset was downloaded. See examples below. Inside the referring paper, these items could be cited as regular citations with the corresponding entry in the reference list of the main paper; as a citation in the Supplementary Material; or as in-line references within the main text, without the corresponding entry in the reference list. The fairest, traceable and thus desirable combination that gives full recognition to publishers and also contributes to the reproducibility and transparency of the study is a full citation in the main paper (green). Download information that can indirectly lead to the full list of the datasets and, therefore, the data publishers is useful although less desirable (yellow). Finally, the remaining combinations are least desirable, as neither data nor their publisher are readily traceable or accountable (red).

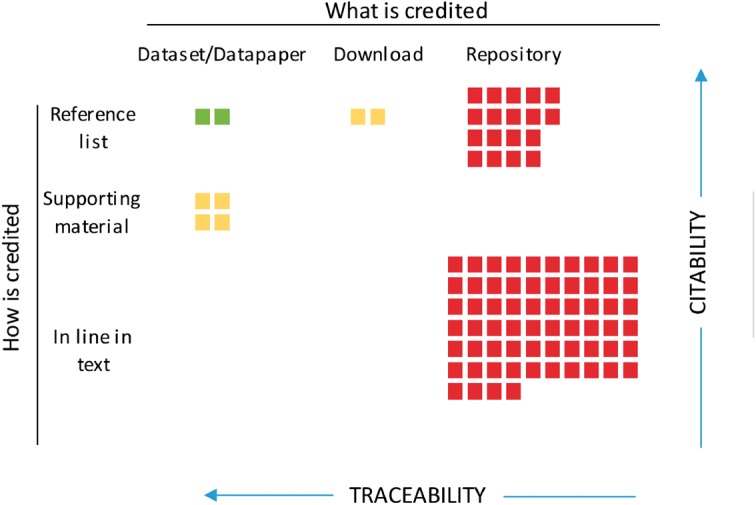

**Figure 1.** Citing practices of the datasets used in the 100 papers revised for the study. Colors represent the desirability of the practice, Green: high, Yellow: medium and Red: low.

Examples of citation practices within the revised papers:

Citation of datasets or data papers: ‘*[…] Observations of xxxx in the Netherlands (Faasse and De Blauwe, 2004; NMR, 2013; Vanagt et al., 2013; Coolen et al., 2015), Belgium (RBINS, 1908; Vanhaelen et al., 2006; VLIZ, 2007; Houziaux et al., 2008; De Blauwe, 2009), […]’*

Citation of reference to the download file: ‘[…] Georeferenced occurrence data with ‘no known coordinate issues’ xxxxxx (doi: 10.15468/dl.g4lis4; n = 20 477 246) and Germany (doi: 10.15468/dl.misihs) […]’

*Generic citation of the repository or aggregator, without reference to the actual dataset: ‘’[…] Fifty-one occurrence records of xxxxx in xxx Province were collected from databases, including field survey data in June 2010 and December 2010, the Global Biodiversity Information Facility (*http://www.gbif.org*) […]’*

1. Costello, M.J., Appeltans,W., Bailly,N. *et al.* (2014) Strategies for the sustainability of online open-access biodiversity databases, *Biol. Conserv.*, **173**, 155–165.

We also retrieved the type of license for all the 13 652 occurrence datasets in GBIF using GBIF’s API (date of access: 21 May 2017). The Attribution license CC-BY 4.0 was the most commonly used license (80.02%), followed by the CC-BY_NC 4.0 (14.13%). Under these licenses, users were actually *required* ‘to attribute the creator of licensed material’ or ‘[to do so] by providing a link to a place where the attribution information may be found’ (30). Therefore, the source of each dataset should be cited in the reference list (preferably) or, at least, be mentioned elsewhere (e.g. in the Supplementary Material or Appendixes). Unfortunately, our pilot study found that only 16 papers (25%) provided some sort of attribution to the original data originators. While it is understandable that for researchers to cite each of the used datasets correctly might have been difficult, the more so for global-scale studies, or about a megadiverse group, or both (31), it should also be noted that GBIF facilitated appropriate means. Along with every data query’s results, either a full list of citable sources (in previous versions of the portal) or a DOI pointing to all contributing datasets was included.

All analysis and data management were performed in R (32) using the following R packages: RefManageR, rcrossref, plyr, readxl, extrafont, bibtex, reshape and ggplot2 (33–36).

## Shared responsibilities of journals and researchers

While data publishing is increasingly being assumed by authors, we believe that such minimal recognition of a major effort (data acquisition, managing, cleaning and sharing) may be a significant obstacle for *quality* data publishing. Journals that require authors to properly cite the digitally accessible knowledge used (DAK, 37) are uncommon. Nevertheless, this might change in the oncoming years. For example, Global Ecology and Biogeography recently announced the creation of a specific reference list section for proper attribution of the datasets used in any meta-analysis or macroecological research. They pointed out that this initiative ‘will ensure that [the data papers] are indexed and get proper citation credit’ (38). We think this is a timely step towards fostering the inclusion of dataset citations by other research journals, which may then be captured by citation services in their metrics.

On the other hand, maybe it is time to stop and rethink if the traditional way of measuring science’s quality, the *impact factor*, will work for datasets (11). This idea has been discussed by taxonomists (39–41), but so far no alternate, readily-usable system seems to have been agreed-upon that may work in the short term to replace citations. For datasets, proposals such as the Citation Usage Index (42) or the data citation index (Thomsom Reuters) have been tabled. Other tools as DataCite have also emerged, focusing on standardizing citation of datasets.

Whatever the procedure, we are convinced that a complete solution requires the participation of authors. It is in the hands of researchers, who use such data, to properly cite the datasets or the associated data papers if they exist. Failure to do so weakens the link between data and discovery and reduces the probability of the latter by jeopardizing the availability of the former. Publishing data is a highly demanding process that not only consists of collating, debugging, standardizing and formatting the data and preparing the metadata, but also includes the previous work required to generate the dataset: broadly, study design, field and laboratory work and digitization. As researchers, we must responsibly acknowledge and credit such significant work contributed by data publishers. However, our pilot research on citation practices among GBIF data users shows that licenses’ terms are generally not being respected, and most of the data publishers remain thus unacknowledged.

Some might think that citing data sources will be an extra burden on researchers, mostly in research that manages multiple taxa at global scales (31). However, we think this will not be necessarily true. For example, GBIF greatly facilitates this task by providing a DOI pointing to each of the downloaded datasets. A widespread use of this strategy across repositories should both significantly lighten the citation task and facilitate automation of the citation tracking. Surprisingly, even though GBIF recommends using this doi to track and cite data publishers, our preliminary study showed that only 27% used this citation practice (8% in the reference list and the rest in Supplementary Material).

Regarding data papers, we believe that a further step might facilitate authors to properly cite resources. It could be advantageous both for data users and data publishers that the metadata for downloaded datasets would include a reference to the corresponding data paper, if it exists. In the case of GBIF, it would be sufficient to allow a further field in the dataset’s metadata to reference the associated data paper, to be updated by the data publisher and providing this field in the citation file. Authors could then cite the data paper easily without having to scour the literature to discover whether the dataset they are reusing was described in a data paper. Failure to credit an existing data paper might be detrimental for the data paper idea from the point of view of recognition, as it would result in undesirably high rates of self-citation (21): only the authors (and perhaps a few thorough researchers) might get to cite the data papers. Moreover, failing to recognize the work of data publishers might lead to a decrease in datasets shared online or, even worst, a drop in the quality of the data shared and therefore, science’s quality.

## Conclusion

Concerns about the recognition of the role of data publishers are rising, and some journals are correspondingly taking stakes (19, 38, 43). If journals start to consistently require proper citation of borrowed data, the issue of unrecognized data publishers shall likely fade away. Nonetheless, we must not forget our responsibility as data users. While no responsible researcher would use freely available data without a proper assessment of its fitness-for-use, the ease with which we can download data from the internet before that assessment work cannot be mistaken for free lunch: even though it may effectively act as a common resource, it has been contributed by other scientists at a significant cost. Failure to recognize that may result in a new tragedy of the commons, where contributions may eventually dwindle as the effort to make them is re-routed to more profitable endeavours. Citing datasets should have the same formal consideration as citing other sources of information, ideas, or previous works. We agree with Chavan (6) in that a cultural change is needed towards professional publication of data, and we posit that peer-recognition of effectively-used, but effortlessly-obtained ‘free’ data is part of such change. This will enhance data publication and give enough incentives to researchers to embrace the open science movement in pursuit of a better understanding of biodiversity and its conservation.

## Supplementary data

Supplementary data are available at *Database* Online.

## Supplementary Material

Supplementary Data
