# Supplementary material for: The tragedy of the biodiversity data commons: a data impediment creeping nigher?
Source: Database (Oxford). 2018 Apr 9;2018:bay033. doi: 10.1093/database/bay033 (PMC5892138; doi:10.1093/database/bay033)
Supplement: Supplementary Data [file bay033_supp.docx]

Appendix S1. List of the 100 papers randomly sourced from the GBIF Public Library Group (11/05/2017) to record the citation practices of the datasets retrieved from GBIF.

Alhajeri, B. H. (2017) Craniomandibular Variation in the Taxonomically Problematic Gerbil Genus Gerbillus (Gerbillinae, Rodentia): Assessing the Influence of Climate, Geography, Phylogeny, and Size, J. Mamm. Evol., 1–16.

Beukhof, E. D., Coolen, J. W. P., van der Weide, B. E., et al. (2016) Records of five bryozoan species from offshore gas platforms rare for the Dutch North Sea, Mar. Biodivers. Rec., 9, 91.

Cornwell, W. K., Westoby, M., Falster, D. S., et al. (2014) Functional distinctiveness of major plant lineages, J. Ecol., 102, 345–356.

Deblauwe, V., Droissart, V., Bose, R., et al. (2016) Remotely sensed temperature and precipitation data improve species distribution modelling in the tropics, Glob. Ecol. Biogeogr., 25, 443–454.

Demos, T. C., Kerbis Peterhans, J. C., Agwanda, B., et al. (2014) Uncovering cryptic diversity and refugial persistence among small mammal lineages across the Eastern Afromontane biodiversity hotspot, Mol. Phylogenet. Evol., 71, 41–54.

Di Febbraro, M., Martinoli, A., Russo, D., et al. (2016) Modelling the effects of climate change on the risk of invasion by alien squirrels, Hystrix, Ital. J. Mammal., 27.

Doudna, J. W. and Danielson, B. J. (2015) Rapid Morphological Change in the Masticatory Structures of an Important Ecosystem Service Provider, PLoS One, 10, e0127218.

Duffy, G. A. and Chown, S. L. (2016) Urban warming favours C4plants in temperate European cities, J. Ecol., 104, 1618–1626.

Escobar, L. E., Qiao, H., Phelps, N. B. D., et al. (2016) Realized niche shift associated with the Eurasian charophyte Nitellopsis obtusa becoming invasive in North America, Sci. Rep., 6, 29037.

Faleiro, F. V., Silva, D. P., de Carvalho, R. A., et al. (2015) Ring out the bells, we are being invaded! Niche conservatism in exotic populations of the Yellow Bells, Tecoma stans (Bignoniaceae), Nat. Conserv., 13, 24–29.

Fitzsimons, M. S. and Miller, R. M. (2009) Serpentine soil has little influence on the root-associated microbial community composition of the serpentine tolerant grass species Avenula sulcata, Plant Soil, 330, 393–405.

Follak, S. and Essl, F. (2013) Spread dynamics and agricultural impact of Sorghum halepense , an emerging invasive species in Central Europe, Weed Res., 53, 53–60.

Fourcade, Y., Engler, J. O., Besnard, A. G., et al. (2013) Confronting expert-based and modelled distributions for species with uncertain conservation status: A case study from the corncrake (Crex crex), Biol. Conserv., 167, 161–171.

García-Roselló, E., Guisande, C., Manjarrés-Hernández, A., et al. (2015) Can we derive macroecological patterns from primary Global Biodiversity Information Facility data?, Glob. Ecol. Biogeogr., 24, 335–347.

González-Santos, R., Cadena-Íñiguez, J., Morales-Flores, F. J., et al. (2017) Prediction of the effects of climate change on Sechium edule (Jacq.) Swartz varietal groups in Mexico, Genet. Resour. Crop Evol., 64, 791–804.

Halama, M., Poliwoda, A., Jasicka-Misiak, I., et al. (2014) Pholiotina cyanopus, a rare fungus producing psychoactive tryptamines, Open Life Sci., 10.

Hannah, L., Ikegami, M., Hole, D. G., et al. (2013) Global Climate Change Adaptation Priorities for Biodiversity and Food Security, PLoS One, 8, e72590.

Hantemirova, E. V, Heinze, B., Knyazeva, S. G., et al. (2016) A new Eurasian phylogeographical paradigm? Limited contribution of southern populations to the recolonization of high latitude populations {inJuniperus} {communisL}.~(Cupressaceae), J. Biogeogr., 44, 271–282.

Harsch, M. A. and HilleRisLambers, J. (2016) Climate Warming and Seasonal Precipitation Change Interact to Limit Species Distribution Shifts across Western North America, PLoS One, 11, e0159184.

Herkt, K. M. B., Barnikel, G., Skidmore, A. K., et al. (2016) A high-resolution model of bat diversity and endemism for continental Africa, Ecol. Modell., 320, 9–28.

Hobbs, C. R. and Baldwin, B. G. (2013) Asian origin and upslope migration of Hawaiian Artemisia (Compositae-Anthemideae), J. Biogeogr., 40, 442–454.

Ihlow, F., Bonke, R., Hartmann, T., et al. (2015) Habitat suitability, coverage by protected areas and population connectivity for the Siamese crocodile Crocodylus siamensis Schneider, 1801, Aquat. Conserv. Mar. Freshw. Ecosyst., 25, 544–554.

Ihlow, F., Dambach, J., Engler, J. O., et al. (2012) On the brink of extinction? How climate change may affect global chelonian species richness and distribution, Glob. Chang. Biol., 18, 1520–1530.

Jaffé, R., Pope, N., Acosta, A. L., et al. (2016) Beekeeping practices and geographic distance, not land use, drive gene flow across tropical bees, Mol. Ecol., 25, 5345–5358.

Jia, L.-B., Huang, Y.-J., Sun, H., et al. (2017) First fossil of Pterolobium (Leguminosae) from the Middle Miocene Yunnan, South China, Rev. Palaeobot. Palynol.

Kanturski, M., Bugaj-Nawrocka, A. and Wieczorek, K. (2016) Pine pest aphids of the genus Eulachnus (Hemiptera: Aphididae: Lachninae): how far can their range extend?, Agric. For. Entomol., 18, 398–408.

Koehler, K., Center, A. and Cavender-Bares, J. (2011) Evidence for a freezing tolerance-growth rate trade-off in the live oaks (Quercus series Virentes) across the tropical-temperate divide, New Phytol., 193, 730–744.

Kozhoridze, G., Orlovsky, N., Orlovsky, L., et al. (2015) Geographic distribution and migration pathways of Pistacia - present, past and future, Ecography (Cop.)., 38, 1141–1154.

Lee-Yaw, J. A., Kharouba, H. M., Bontrager, M., et al. (2016) A synthesis of transplant experiments and ecological niche models suggests that range limits are often niche limits, Ecol. Lett.

Lessmann, J., Guayasamin, J. M., Casner, K. L., et al. (2016) Freshwater vertebrate and invertebrate diversity patterns in an Andean-Amazon basin: implications for conservation efforts, Neotrop. Biodivers., 2, 99–114.

Liede-Schumann, S., Khanum, R., Mumtaz, A. S., et al. (2016) Going west – A subtropical lineage ( Vincetoxicum , Apocynaceae: Asclepiadoideae) expanding into Europe, Mol. Phylogenet. Evol., 94, 436–446.

Liedtke, H. C., Müller, H., Hafner, J., et al. (2017) Terrestrial reproduction as an adaptation to steep terrain in African toads, Proc. R. Soc. B Biol. Sci., 284, 20162598.

Linder, H. P., Antonelli, A., Humphreys, A. M., et al. (2013) What determines biogeographical ranges? Historical wanderings and ecological constraints in the danthonioid grasses, J. Biogeogr., 40, 821–834.

Lira-Noriega, A. and Peterson, A. T. (2014) Range-wide ecological niche comparisons of parasite, hosts and dispersers in a vector-borne plant parasite system, J. Biogeogr., 41, 1664–1673.

Lira-Noriega, A., Soberón, J. and Miller, C. P. (2013) Process-based and correlative modeling of desert mistletoe distribution: a multiscalar approach, Ecosphere, 4, art99.

Liu, H. and Osborne, C. P. (2014) Water relations traits of C4 grasses depend on phylogenetic lineage, photosynthetic pathway, and habitat water availability., J. Exp. Bot., eru430-.

Lübcker, N., Zengeya, T., Dabrowski, J., et al. (2014) Predicting the potential distribution of invasive silver carp Hypophthalmichthys molitrix in South Africa, African J. Aquat. Sci., 39, 157–165.

Lucifora, L. O., García, V. B. and Worm, B. (2011) Global Diversity Hotspots and Conservation Priorities for Sharks, PLoS One, 6, e19356.

Magwé-Tindo, J., Zapfack, L. and Sonké, B. (2016) Diversity of wild yams (Dioscorea spp., Dioscoreaceae) collected in continental Africa, Biodivers. Conserv., 25, 77–91.

Malhado, A. C. M., Oliveira-Neto, J. A., Stropp, J., et al. (2015) Climatological correlates of seed size in Amazonian forest trees, J. Veg. Sci., 26, 956–963.

Mendoza-Fernández, A. J., Pérez-García, F. J., Martínez-Hernández, F., et al. (2015) Areas of endemism and threatened flora in a Mediterranean hotspot: Southern Spain, J. Nat. Conserv., 23, 35–44.

Merow, C., Allen, J. M., Aiello-Lammens, M., et al. (2016) Improving niche and range estimates with Maxent and point process models by integrating spatially explicit information, Glob. Ecol. Biogeogr.

Merow, C., Bois, S. T., Allen, J. M., et al. (2017) Climate change both facilitates and inhibits invasive plant ranges in New England, Proc. Natl. Acad. Sci., 201609633.

Meyer, A. L. S., Pie, M. R. and Passos, F. C. (2013) Assessing the exposure of lion tamarins (Leontopithecusspp.) to future climate change, Am. J. Primatol., 76, 551–562.

Miller, J. S., Krupnick, G. A., Stevens, H., et al. (2013) Toward Target 2 of the Global Strategy for Plant Conservation: An Expert Analysis of the Puerto Rican Flora to Validate New Streamlined Methods for Assessing Conservation Status1, Ann. Missouri Bot. Gard., 99, 199–205.

Molina-Henao, Y. F., Guerrero-Chacón, A. L. and Jaramillo, M. A. (2016) Ecological and Geographic Dimensions of Diversification in <I>Piper</I> subgenus <I>Ottonia</I>: A Lineage of Neotropical Rainforest Shrubs, Syst. Bot., 41, 253–262.

Multimäki, S., Hall, A. and Ahonen-Rainio, P. (2016) Comparison of Temporally Classified and Unclassified Map Animations, Cartogr. Perspect.

Nicolas, V., Martínez-Vargas, J. and Hugot, J.-P. (2016) Molecular data and ecological niche modelling reveal the evolutionary history of the common and Iberian moles (Talpidae) in Europe, Zool. Scr.

Ortiz-Rodriguez, A. E., Burelo Ramos, C. M. and Gomez-Dominguez, H. (2016) A new species of Amphitecna (Bignoniaceae) endemic to Chiapas, Mexico, PhytoKeys, 65, 15–23.

Osipova, L. and Sangermano, F. (2016) Surrogate species protection in Bolivia under climate and land cover change scenarios, J. Nat. Conserv., 34, 107–117.

Ostrowski, M.-F., Prosperi, J.-M. and David, J. (2016) Potential Implications of Climate Change on Aegilops Species Distribution: Sympatry of These Crop Wild Relatives with the Major European Crop Triticum aestivum and Conservation Issues., PLoS One, 11, e0153974.

Padonou, E. A., Teka, O., Bachmann, Y., et al. (2015) Using species distribution models to select species resistant to climate change for ecological restoration ofbowéin West Africa, Afr. J. Ecol., 53, 83–92.

Paine, C. E. T., Stahl, C., Courtois, E. A., et al. (2010) Functional explanations for variation in bark thickness in tropical rain forest trees, Funct. Ecol., 24, 1202–1210.

Perktaş, U., Gür, H., Sağlam, İ. K., et al. (2015) Climate-driven range shifts and demographic events over the history of Kruper’s Nuthatch Sitta krueperi, Bird Study, 62, 14–28.

Pickles, R. S. A., Thornton, D., Feldman, R., et al. (2013) Predicting shifts in parasite distribution with climate change: a multitrophic level approach, Glob. Chang. Biol., 19, 2645–54.

Pyle, R. L., Boland, R., Bolick, H., et al. (2016) A comprehensive investigation of mesophotic coral ecosystems in the Hawaiian Archipelago, PeerJ, 4, e2475.

Qin, Z., Zhang, J.-E., DiTommaso, A., et al. (2015) Predicting invasions of Wedelia trilobata (L.) Hitchc. with Maxent and GARP models, J. Plant Res., 128, 763–775.

Ramsey, J. M., Peterson, A. T., Carmona-Castro, O., et al. (2015) Atlas of Mexican Triatominae (Reduviidae: Hemiptera) and vector transmission of Chagas disease., Mem. Inst. Oswaldo Cruz, 110, 339–52.

Record, S., Charney, N. D., Zakaria, R. M., et al. (2013) Projecting global mangrove species and community distributions under climate change, Ecosphere, 4, art34.

Reginato, M. and Michelangeli, F. A. (2016) Diversity and constraints in the floral morphological evolution of Leandra s.str. (Melastomataceae), Ann. Bot., 118, 445–458.

Ribeiro, V., Peterson, A. T., Werneck, F. P., et al. (2016) Ecological and historical views of the diversification of Geositta miners (Aves: Furnariidae: Sclerurinae), J. Ornithol., 1–9.

Rocchini, D., Garzon-Lopez, C. X., Marcantonio, M., et al. (2017) Anticipating species distributions: Handling sampling effort bias under a Bayesian framework, Sci. Total Environ.

Rodrigues, E. S. da C., Rodrigues, F. A., Rocha, R. L. de A. da, et al. (2010) Evaluation of different aspects of maximum entropy for niche-based modeling, Procedia Environ. Sci., 2, 990–1001.

Sanín, M. J., Kissling, W. D., Bacon, C. D., et al. (2016) The Neogene rise of the tropical Andes facilitated diversification of wax palms ( Ceroxylon : Arecaceae) through geographical colonization and climatic niche separation, Bot. J. Linn. Soc., 182, 303–317.

Sardari, S., Shokrgozar, M. A. and Ghavami, G. (2009) Cheminformatics based selection and cytotoxic effects of herbal extracts, Toxicol. Vitr., 23, 1412–1421.

Särkinen, T., Iganci, J. R., Linares-Palomino, R., et al. (2011) Forgotten forests - issues and prospects in biome mapping using Seasonally Dry Tropical Forests as a case study, BMC Ecol., 11, 27.

Sarma, R. R., Munsi, M. and Ananthram, A. N. (2015) Effect of Climate Change on Invasion Risk of Giant African Snail (Achatina fulica Férussac, 1821: Achatinidae) in India, {PLOS} {ONE}, 10, e0143724.

Schleuning, M., Fründ, J., Schweiger, O., et al. (2016) Ecological networks are more sensitive to plant than to animal extinction under climate change, Nat. Commun., 7, 13965.

Schrautzer, J., Fichtner, A., Huckauf, A., et al. (2011) Long-term population dynamics of Dactylorhiza incarnata (L.) Soó after abandonment and re-introduction of mowing, Flora - Morphol. Distrib. Funct. Ecol. Plants, 206, 622–630.

Schuettpelz, E., Pryer, K. M. and Windham, M. D. (2015) A Unified Approach to Taxonomic Delimitation in the Fern Genus <I>Pentagramma</I> (Pteridaceae), Syst. Bot., 40, 629–644.

Selama, O., James, P., Nateche, F., et al. (2013) The World Bacterial Biogeography and Biodiversity through Databases: A Case Study of NCBI Nucleotide Database and GBIF Database, Biomed Res. Int., 2013, 1–11.

Senczuk, G., Colangelo, P., De Simone, E., et al. (2017) A combination of long term fragmentation and glacial persistence drove the evolutionary history of the Italian wall lizard Podarcis siculus, BMC Evol. Biol., 17, 6.

Shabani, F. and Kumar, L. (2013) Risk Levels of Invasive Fusarium oxysporum f. sp. in Areas Suitable for Date Palm (Phoenix dactylifera) Cultivation under Various Climate Change Projections, PLoS One, 8, e83404.

Shabani, F., Kumar, L. and Taylor, S. (2015) Distribution of date palms in the middle east based on future climate scenarios, Exp. Agric., 51, 244–263.

Shaik, R. S., Burrows, G. E., Urwin, N. A. R., et al. (2017) The biology and management of prickly paddy melon (Cucumis myriocarpus L.), an important summer annual weed in Australia, Crop Prot., 92, 29–40.

Sosa, V., Ornelas, J. F., Ramírez-Barahona, S., et al. (2016) Historical reconstruction of climatic and elevation preferences and the evolution of cloud forest-adapted tree ferns in Mesoamerica, PeerJ, 4, e2696.

Steadman, D. W. and Franklin, J. (2014) Changes in a West Indian bird community since the late Pleistocene, J. Biogeogr., 42, 426–438.

Stewart, J. A. E., Perrine, J. D., Nichols, L. B., et al. (2015) Revisiting the past to foretell the future: Summer temperature and habitat area predict pika extirpations in California, J. Biogeogr., 42, 880–890.

Stropp, J., Ladle, R. J., Ana, A. C., et al. (2016) Mapping ignorance: 300 years of collecting flowering plants in Africa, Glob. Ecol. Biogeogr., 25, 1085–1096.

Stuart-Smith, R. D., Edgar, G. J., Barrett, N. S., et al. (2015) Thermal biases and vulnerability to warming in the world’s marine fauna, Nature, advance on.

Sutkowska, A., Pasierbiński, A., Warzecha, T., et al. (2014) Multiple cryptic refugia of forest grass Bromus benekenii in Europe as revealed by ISSR fingerprinting and species distribution modelling, Plant Syst. Evol., 300, 1437–1452.

Taylor, S., Kumar, L. and Reid, N. (2012) Impacts of climate change and land-use on the potential distribution of an invasive weed: a case study of Lantana camara in Australia, Weed Res., 52, 391–401.

Taylor, S. and Kumar, L. (2014) Climate Change and Weed Impacts on Small Island Ecosystems: Lantana camara L. (Magnoliopsida: Verbenaceae) Distribution in Fiji1, Pacific Sci., 68, 117–133.

Taylor, S., Kumar, L., Reid, N., et al. (2012) Climate Change and the Potential Distribution of an Invasive Shrub, Lantana camara L, PLoS One, 7, e35565.

Tererai, F. and Wood, A. R. (2014) On the present and potential distribution of Ageratina adenophora (Asteraceae) in South Africa, South African J. Bot., 95, 152–158.

Thesing, B. D., Noyes, R. D., Starkey, D. E., et al. (2015) Pleistocene climatic fluctuations explain the disjunct distribution and complex phylogeographic structure of the Southern Red-backed Salamander, Plethodon serratus, Evol. Ecol.

Trethowan, P. D., Robertson, M. P. and McConnachie, A. J. (2011) Ecological niche modelling of an invasive alien plant and its potential biological control agents, South African J. Bot., 77, 137–146.

Valle, M., Chust, G., del Campo, A., et al. (2014) Projecting future distribution of the seagrass Zostera noltii under global warming and sea level rise, Biol. Conserv., 170, 74–85.

Velez\textendashLiendo, X., Strubbe, D. and Matthysen, E. (2013) Effects of variable selection on modelling habitat and potential distribution of the Andean bear in Bolivia, Ursus, 24, 127–138.

Wetterer, J. K. (2011) Worldwide Spread {ofPheidole} teneriffana(Hymenoptera: Formicidae), Florida Entomol., 94, 843–847.

Willis, C. G., Franzone, B. F., Xi, Z., et al. (2014) The establishment of Central American migratory corridors and the biogeographic origins of seasonally dry tropical forests in Mexico, Front. Genet., 5.

Wyse, S. V and Dickie, J. B. (2017) Predicting the global incidence of seed desiccation sensitivity, J. Ecol., 105, 1082–1093.

Yang, J., Di, X., Meng, X., et al. (2016) Phylogeography and evolution of two closely related oak species (Quercus) from north and northeast China, Tree Genet. Genomes, 12, 89.

Yessoufou, K., Daru, B. H., Tafirei, R., et al. (2017) Integrating biogeography, threat and evolutionary data to explore extinction crisis in the taxonomic group of cycads, Ecol. Evol.

Yi, Y., Cheng, X., Yang, Z.-F., et al. (2016) Maxent modeling for predicting the potential distribution of endangered medicinal plant (H. riparia Lour) in Yunnan, China, Ecol. Eng., 92, 260–269.

Zhao, M., Alström, P., Hu, R., et al. (2016) Phylogenetic relationships, song and distribution of the endangered Rufous-headed {RobinLarvivora} ruficeps, Ibis (Lond. 1859)., 159, 204–216.

Zhao, X.-L., Gao, X.-F., Zhu, Z.-M., et al. (2017) The demographic response of a deciduous shrub (the Indigofera bungeana complex, Fabaceae) to the Pleistocene climate changes in East Asia, Sci. Rep., 7, 697.

Zhu, G.-P., Rédei, D., Kment, P., et al. (2013) Effect of geographic background and equilibrium state on niche model transferability: predicting areas of invasion of Leptoglossus occidentalis, Biol. Invasions, 16, 1069–1081.

Zigouris, J., Schaefer, J. A., Fortin, C., et al. (2013) Phylogeography and Post-Glacial Recolonization in Wolverines (Gulo gulo) from across Their Circumpolar Distribution, PLoS One, 8, e83837.

Zurano, J. P., Martinez, P. A., Canto-Hernandez, J., et al. (2017) Morphological and ecological divergence in South American canids, J. Biogeogr., 44, 821–833.
